# Gaze shifts during wayfinding decisions

**DOI:** 10.3758/s13414-023-02797-z

**Published:** 2023-10-18

**Authors:** Mai Geisen, Otmar Bock, Stefanie Klatt

**Affiliations:** Institute of Exercise Training and Sport Informatics, German Sport University Cologne, Am Sportpark Müngersdorf 6, 50933 Cologne, Germany

**Keywords:** Decision-making, Eye movements, Navigation

## Abstract

When following a route through a building or city, we must decide at every intersection in which direction to proceed. The present study investigates whether such decisions are preceded by a gradual gaze shift in the eventually chosen direction. Participants were instructed to repeatedly follow a route through a sequence of rooms by choosing, in each room, the correct door from among three possible doors. All rooms looked alike, except for a room-specific cue, which participants could associate with the direction to take. We found that on 88.9% of trials, the gaze shifted from the cue to the chosen door by a single saccade, *without* interim fixations. On the few trials where interim fixations occurred, their spatiotemporal characteristics differed significantly from that expected in case of a consistent shift. Both findings concordantly provide no support for the hypothesized gradual gaze shift. The infrequent interim fixations might rather serve the purpose to avoid large-amplitude saccades between cue and door.

## Introduction

Finding our way through a building or a city is a highly complex cognitive task. It involves the integration of spatial cues from multiple sensory modalities, the formation of egocentric and allocentric representations of space, decision-making, route planning, locomotor control, and the executive coordination of these processes (Hegarty et al., 2022; Wolbers & Hegarty, 2010). The decision-making component of wayfinding has been investigated with gaze registrations, which offer an unobtrusive way to assess the allocation of attention, even though gaze and attention can be dissociated (for a review, see, e.g., Henderson, 2003). In that research, participants saw an intersection with several departing passageways and had to decide along which passageway to proceed. Gaze registrations revealed that shortly before an overt decision was made, the participants’ gaze was increasingly attracted towards the chosen side (Geisen et al., 2021; Wiener et al., 2011, 2012). Specifically, those authors subdivided an interval that started either 1.5 s (Wiener et al., 2011), or 2 s (Wiener et al., 2012), or 4 s before the overt decision (Geisen et al., 2021) into bins of either 50 ms width (Wiener et al., 2011, 2012) or 200 ms width (Geisen et al., 2021). They then calculated for each bin the incidence of fixations on the eventually chosen side rather than the opposite side. In all studies, that incidence was about 50% for early bins, and gradually increased to about 80% thereafter (i.e., within the last 1–2 s before the decision was made). This increasing likelihood of looking at the eventually chosen side was called “gaze bias.”

An increasing gaze bias towards the eventually chosen side is not specific for wayfinding decisions. In other decision-making tasks, for instance when selecting the more attractive among two concurrently displayed paintings, the likelihood of looking at the chosen item also increased gradually from chance level to about 80% within the last second before the overt decision was made (Fiedler & Glöckner, 2012; Glaholt & Reingold, 2009; Shimojo et al., 2003). This gaze bias was not interrupted when the items were removed, which confirms that it is related to decision-making rather than to visual processing (Simion & Shimojo, 2007).

It is important to note that in past research on decision-making, the gaze bias was quantified as the proportion of time that participants looked at the selected location rather than at other location(s). This leaves open whether participants moved their eyes to the eventually selected location gradually, with one or two fixations along the way, or rather moved their eyes to the selected location abruptly, with a single saccade, and the gradual gaze bias emerged only by averaging across trials with different saccade onset times. For example, a gaze bias of 50% at 300 ms before a decision could indicate that at this time, participants looked mid-way between the initial and the eventually selected location; however, it could also indicate that at this time, half of participants looked at the initial location, and the other half looked at the eventually selected location.

It might seem unlikely that the gaze would shift to the eventually selected location gradually, with interim fixations along the way, since past research consistently observed that the gaze shifts to visual stimuli either by a single saccade, or by a primary saccade followed by a small corrective saccade (e.g., Becker, 1972). The corrective saccade covers only about 5% to 10% of total stimulus distance, both when a single stimulus is presented (Henson, 1978; Weber & Daroff, 1972) and when a realistic visual scene is displayed (Unema et al., 2005; Zelinsky et al., 1997). However, this research dealt with stimulus detection or with visual inspection, and gaze behavior may be different when decisions are made in a wayfinding task, possibly because the cognitive task demand is higher. The existence of a gradual gaze shift in decision-making would therefore be of general theoretical interest: it would indicate that the principles of gaze control may vary in dependence on the underlying behavioral task. We addressed this issue by asking participants to make wayfinding decisions, and by evaluating *not* the likelihood of looking in the eventually chosen direction, but rather the actual spatiotemporal fixation pattern. We reasoned that, if the gaze shifted to the eventually chosen location gradually, the majority of trials should exhibit interim fixations that reflect neither inaccurate saccades nor deliberate inspections of items elsewhere in the display. We further reasoned that those interim fixations should retrace a continuous shift towards the selected location, so that fixations occurring later in time would be located closer to that location.

To carry out this work, we took advantage of an ongoing research project, designed to explore a new wayfinding paradigm. In that paradigm, participants follow a prescribed route through a flight of 30 rooms by choosing, in each room, the correct door from among three possible doors. All rooms look alike and contain very few visual details, except for a distinctive visual cue on the floor, which is different in each room. Participants therefore can identify each room by that cue. In wayfinding literature, cues that help travelers to find their way are typically called “landmarks,” and we will therefore call the cues in our study landmarks as well. In analogy to earlier work on wayfinding (Cohen & Schuepfer, 1980; Geisen et al., 2021; Wiener et al., 2011, 2012), we used an experimental design that isolates decision-making from other cognitive processes that occur in wayfinding, such as self-motion monitoring, route planning and locomotor control. Participants viewed a still scene, decided which direction to take, and are were then transferred to the next still scene without real or simulated travel in between. For the purposes of the present research, we took advantage of this validation project and analyzed gaze data that were registered during two trips along a prescribed route.

## Method

### Participants and procedures

Data were collected from 20 participants (24.25 ± 3.45 years of age, four females, 16 males) in the second half of the year 2021. Most of them were recruited on campus. All had a college or university entrance qualification, reported to be free of neurological and psychiatric diseases, and had normal or corrected to normal vision. Approval was obtained from a German ethics board. All participants gave written informed consent before testing began.

A power analysis was conducted for the two statistical tests of primary interest, using G*Power (Faul et al., 2007). As detailed below, one was a test of a single proportion against the fixed value of 0.5, applied to all gaze shifts towards the eventually selected door; using *g* = 0.3 (i.e., a medium-sized effect), α = 0.05 and 1 − ß = 0.95, we yielded *n* = 35. The other was a test of unit slope, applied to all interim fixations during those gaze shifts; using ρ1 = 1, ρ0 = 0, α = 0.05 and 1 − ß = 0.95, we yielded *n* = 134. Since we analyzed 1,200 decisions (20 participants × 2 trips × 30 rooms, see below) and assumed that interim fixations will occur frequently, we anticipated to yield much more than 35 gaze shifts and 134 interim fixations. Actually, however, interim fixations were quite rare and we therefore could register only 111. Nevertheless, since statistical significance was very high (*p* < .001, see below), we are confident that the experimentally determined slope of 0.04 indeed differs from a slope of 1.00.

In a single testing session of about one hour, participants signed the informed consent statement, then completed a demographics questionnaire, and then performed our wayfinding task. This study was not preregistered.

### Transparency and openness

We adhered to the journal’s transparency and openness promotion guidelines. The results of the study are limited in terms of generality.

### Wayfinding task

**Fig. 1:**
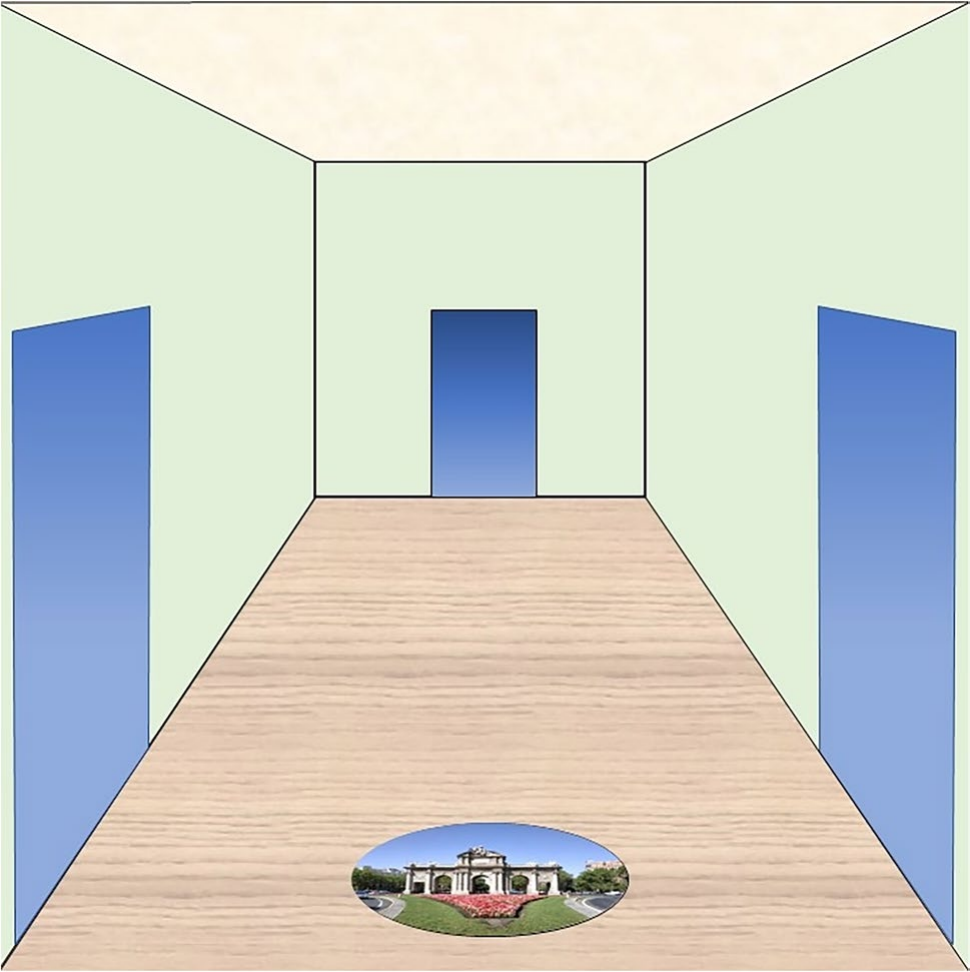
Example of a room in the wayfinding task. The size, shape, location, color and patterning of all room elements was the same in each room, except for the room-specific photographs. Participants were told that the blue elements represent doors. (Color figure online)

Participants were seated in front of a 24-in. computer monitor (1,024 × 768 pixels), located at eye level, about 60 cm ahead. The monitor displayed a sequence of thirty still images, each showing the outline of a room with a left, a center and a right door, and an oval photograph on the floor (cf. Fig. 1). All rooms looked exactly the same, except for the photograph, which showed a different motif in each room: a landscape, building, animal, plant or artistic painting. In what follows, we will refer to those photographs as “landmarks.”

Participants were instructed to follow a prescribed route through all thirty rooms by selecting the correct door in each room. Upon presentation of a given room, they had to indicate through which door they intend to proceed by moving the cursor of a computer mouse from the landmark to the chosen door, and then clicking the mouse button. There was no time limit for responding. If participants clicked on the correct door, a success message appeared, and participants then clicked a “continue” button to display the next room. If participants clicked anywhere outside the correct door, an error message appeared; participants had to click the “continue” button, the cursor was then displaced across the monitor, participants had to bring the cursor back and click again; the previous room was then displayed once more, and participants could choose another door. The double-clicking procedure was designed to be inconvenient, and thus, to discourage participants from randomly clicking at the doors until they find the correct one by chance.

The first trip through the 30 rooms was experimenter guided: in each room, the experimenter announced which door to choose, and the participant then clicked on that door. The second to eighth trip was self-guided: the participant decided which door to choose, and then clicked on that door. The sequence of landmarks and associated doors was the same in all eight trips, such that the participants could use and enhance their knowledge about landmark–direction associations. After a block of eight trips, participants completed a second, and then a third block of eight trips. Each block represented a different route through the 30 rooms (i.e., a different set of 30 landmarks and a different sequence of direction choices). That sequence was generated for each block quasirandomly, under the constraint that no direction occurred more than twice in a row, and that each direction occurred exactly ten times in total.

The participants’ gaze position was registered with a head-mounted, video-based gaze tracking system (Pupil Core binocular, Pupil Labs GmbH, weight: 23g). The eye image was scanned at 200 Hz with a resolution of 192 × 192 pixel, and the visual scene at 60 Hz with a resolution of 720 × 720 pixel. The software Pupil Capture (v3.2.20) was used for 5-point calibration and subsequent gaze registration. The software Pupil Player (v3.0.7) was used later on to calculate normalized fixation positions, with *x* = 0, *y* = 0 representing the bottom left corner of the computer monitor, and *x* = 1, *y* = 1 representing the top right corner.

### Data analysis

The first two blocks were considered as practice, and the data from the second and the eighth trip of the third block were analyzed. Behavioral performance was quantified as the number of rooms in which participants clicked on the correct door right away, without first clicking at a wrong door in the same room. This number could principally range between 0 and 30 on each trip.

**Fig. 2:**
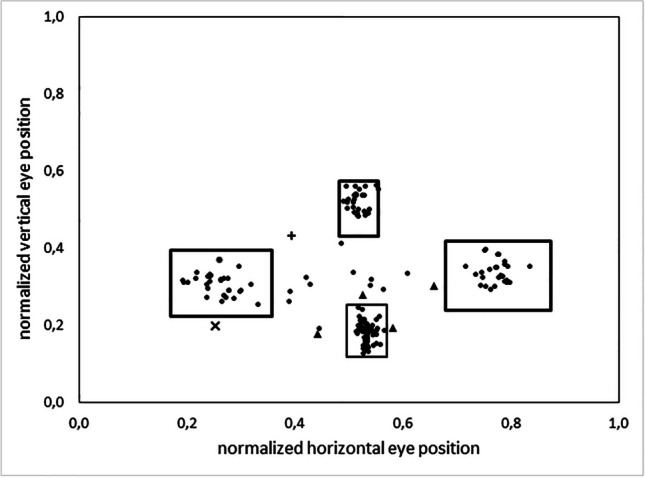
Exemplary plot of fixations and regions of interest for one trip of one participant. Each symbol represents one fixation. The bottom box represents the region of interest (ROI) for the landmark, and the left, central and right boxes represent the ROIs for the three doors. Since the landmark and the doors were displayed in the lower 60% of the screen (cf. Fig. 1), the vertical component of fixations does not exceed 0.6. The symbol ● denotes fixations within a ROI, as well as fixations elsewhere in the visual space that did not occur during gaze shifts from the landmark to the correct door. Fixations that occurred during gaze shifts from the landmark to the correct door are marked as ▲ if they meet our two criteria for interim fixations (cf. main text), by **+** if they do not meet the first criterion, and by **x** if they do not meet the second criterion

To overcome the inherent systematic errors of video-based gaze trackers (overview in Blignaut & Wium, 2014), authors commonly use a priori knowledge to adjust their registered data (e.g., Hornof & Halverson, 2002; Hyrskykari, 2006). For example, the endpoint of a large leftward gaze movement with a small downward component is interpreted as fixation of the first word on a new line (Hyrskykari, 2006). Accordingly, we interpreted gaze positions that cluster at the bottom center of the visual space as fixations on the landmark, and those that cluster in a left, central, or right area of visual space as fixations of the left, central or right door, respectively. In particular, we generated a plot of all fixations from a given participant on a given trip; we then manually drew approximate boundaries around the four fixation clusters, disregarding occasional outliers; an algorithm then calculated the four regions of interest (ROIs) as extending ±3 standard deviations from the center of gravity of each specified area. Standard deviations were calculated separately for the horizontal and the vertical dimension of each ROI, trip, and participant, and were based on all data points from the manually defined cluster. The ROIs thus yielded never overlapped. Fixations within the bottom left, central or right ROI were interpreted as fixations on the landmark or on the left, central, or right door, respectively. Gaze positions outside the four ROIs were interpreted as fixations elsewhere in the visual space. Figure 2 illustrates the outcome of this algorithmic procedure.

For an overview of gaze behavior in our wayfinding task, we calculated several parameters for each participant and trip. If a room was presented repeatedly because the participant’s response was incorrect, only the last repetition (i.e., with the correct response) was considered. The parameters were therefore derived from one presentation of each of our 30 rooms. The following parameters were calculated:

- Total gaze time (TotalT): cumulative duration of all fixations at the 30 displayed rooms. This parameter represents the time during which participants could process visual information about the rooms. It does not include the duration of saccades, the duration of fixations outside the computer monitor, and the duration of feedback messages presented between rooms (see Henderson, 2003).
- Landmark fixation time (LandmarkT): cumulative duration of all fixations at the landmark (see Droll et al., 2020; Wang et al., 2012).
- Correct door fixation time (DoorT): cumulative duration of all fixations at the subsequently selected door (see Brouwer et al., 2021; Castelhano et al., 2009).
- Elsewhere fixation time (ElseT): cumulative duration of all fixations elsewhere in the room. This included fixations at the wrong doors as well as fixations outside the four ROIs (see Tatler, 2007; Zelinsky et al., 2013).

Our main analysis dealt with gaze shifts from the landmark to the correct door, thus considering again only room presentations with a correct response. We defined direct gaze shifts as a sequence of two fixations, the first within the landmark ROI and the second within the correct-door ROI. Furthermore, we defined indirect gaze shifts as a sequence of three or four fixations, the first within the landmark ROI, the last within the correct-door ROI, and the remaining one or two fixations elsewhere in visual space. Those elsewhere fixations were classified as *interim fixations* if they met two criteria: (1) the route from the landmark fixation via the elsewhere fixation(s) to the door fixation should not be longer than 130% of the Euclidian distance between the landmark and the door fixation; (2) the route from the landmark fixation to the elsewhere fixation should not be longer than 90%, and not shorter than 10%, of the Euclidian distance between the landmark fixation and the door fixation. Criterion (1) rejected interim inspections of room elements such as edges, corners or floor details, and criterion (2) rejected fixations prior to small corrective saccades.

When the above definitions were applied to the exemplary data shown in Fig. 2, only six elsewhere fixations were identified. Four of them satisfied the two criteria for interim fixations (▲), one didn’t meet the first criterion (+) and one didn’t meet the second criterion (x). The following parameters were calculated for all identified interim fixations:

normalized interim fixation time (InterimT): interval between landmark fixation and the subsequent interim fixation, divided by the interval between landmark fixation and the first subsequent correct-door fixation,

normalized interim fixation distance (InterimD): Euclidian distance between landmark fixation and the subsequent interim fixation, divided by the Euclidian distance between landmark fixation and the first subsequent correct-door fixation.

## Results

**Fig. 3:**
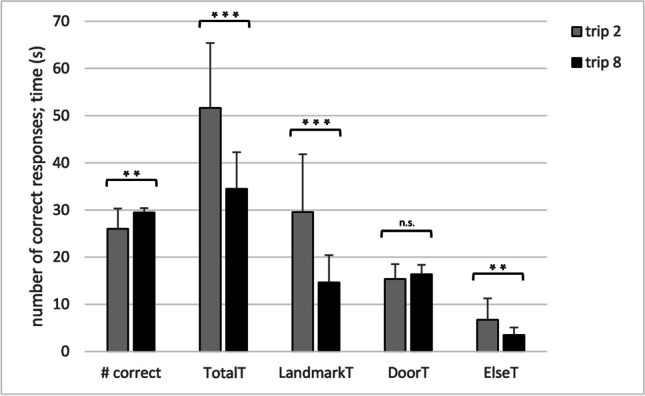
Response accuracy and gaze control in the wayfinding task. Grey bars represent means across participants on the second trip, and black bars the corresponding means on the eighth trip. Error indicators are the between-participant standard deviations. The number of correct responses (# correct) is cumulated across all 30 rooms of the route, the other parameters are defined in Methods. The symbols ***, **, and n.s. denote the significance levels *p* < .001, *p* < .01, and *p* > .05, respectively

Figure 3 shows that participants learned the wayfinding task well: the number of rooms with correct decisions was already high on the second trip, and increased even further from the second to the eighth trip, *t*(19) = 3.642, *p* = .002, *d* = 0.814. Figure 3 further illustrates that this increase of accuracy was accompanied by a decrease of cumulative fixation times, specifically, a decrease of TotalT, *t*(19) = 6.403, *p* < .001, *d* = 1.432; LandmarkT, *t*(19) = 6.016, *p* < .001, *d* = 1.345; and ElseT, *t*(19) = 3.193; *p* = .005, *d* = 0.714. However, DoorT remained quite stable across trips, *t*(19) = 1.429, *p* = 0.169, *d* = 0.320.

We registered a total of 979 gaze shifts from the landmark to the correct door. Among them, 870 were accomplished by a single saccade, and the remaining 109 with one (*n* = 107) or two (*n* = 2) interim fixations. The incidence of gaze shifts with interim fixations therefore was 11.1%, which is significantly different from an incidence of 50% (test of a proportion: *z* = 24.32, *p* < .001). This constitutes firm statistical evidence *against* the view that the majority of trials exhibit interim fixations.

**Fig. 4:**
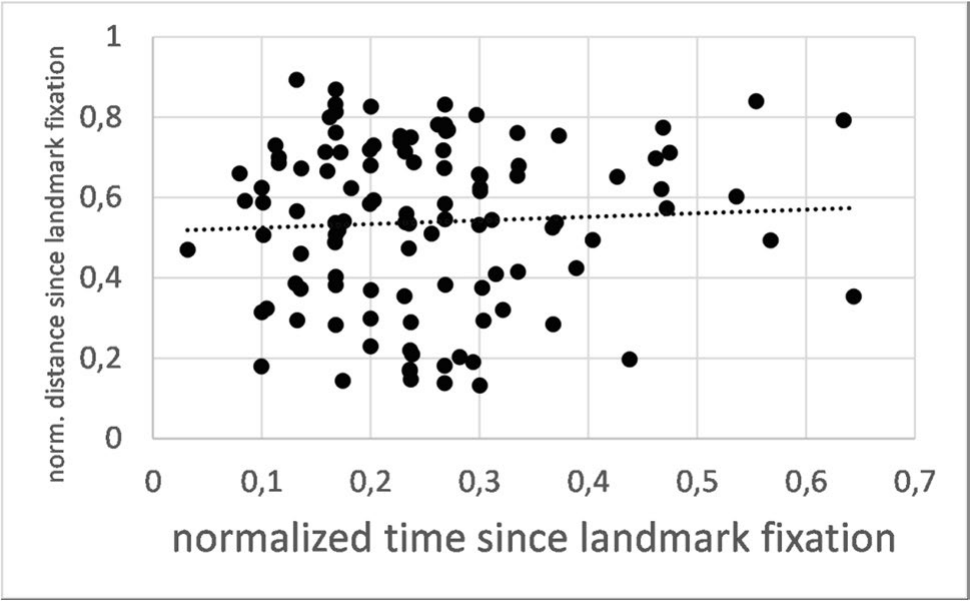
Distance-to-time characteristic of interim fixations. Scores are normalized with respect to the time or distance to the first correct-door fixation. Each dot represents one interim fixation, and the dashed line is the regression line. The regression line is shown only to visualize the general trend, it is not used for statistical inference (cf. Results section)

If interim fixations reflected a continuous gaze shift towards the correct door, their normalized distance should be monotonically related to their normalized time. If so, then the ranks of InterimD and InterimT should be significantly correlated, and the regression slope of those ranks should be close to 1.0. In contrast to this prediction, however, the rank correlation between InterimD and InterimT was not significant (Kendall’s tau = 0.03, *Z* = 0.43, *p* = .667), and the regression slope of the ranks was 0.04, which is significantly different from 1.00, *t*(109) = 10.06, *p* < .001. This constitutes firm statistical evidence against a monotonous relationship, and thus against the view that interim fixations retrace a gradual shift from the landmark to the correct door. Figure 4 accordingly illustrates that InterimD was quite independent of InterimT. Mean ± standard deviation of InterimT were 0.52 ± 0.15, and those of InterimD were 0.54 ± 0.20.

## Discussion

The present study dealt with decision-making in a wayfinding task. Participants had to proceed through a sequence of rooms by choosing one of three doors in each room, and we evaluated whether their decisions might involve a gradual gaze shift towards the eventually chosen door. We predicted that, if this were the case, (1) the majority of gaze shifts towards the door should exhibit interim fixations, and (2) position and distance of those interim fixations should be monotonically related.

An overview of the registered data indicated that participants learned the wayfinding task well: decision accuracy increased, and total gaze time decreased across trips. The decrease of total gaze time was mainly driven by shorter landmark inspections rather than by shorter looks at the correct door. This is in line with our earlier study (Geisen et al., 2021), where total gaze time also decreased across wayfinding trips, while the incidence of fixations on the eventually chosen side did not decrease. A possible interpretation is that total gaze time includes the processing of visual information, which may become more efficient with practice (e.g., Bays & Husain, 2021; Eisma & de Winter, 2020; Thomas & Scharinger, 2022), while looking in the chosen direction reflects mainly the verification of one’s tentative decisions, which may not be practice dependent (e.g., Ghozlan & Widlocher, 1987; Kuhbandner et al., 2010; Reisenzein & Studtmann, 2021).

More importantly, we obtained firm statistical evidence *against* the existence of a gradual gaze shift. Interim fixations did *not* occur on a majority of trials, and those that occurred exhibited *no* monotonous relationship between position and distance. Both findings argue independently against a gradual shift. From this, we conclude that the gaze bias reported in earlier wayfinding literature (see Introduction) is probably an artifact of averaging: The incidence of fixations in the eventually chosen direction probably increased not because the gaze shifted there gradually, but because the gaze shifted there abruptly but at different times on different trials.

It is unlikely that the occasional interim fixations in our study represent inspections of objects elsewhere in the room, since those were rejected by our 130% criterion, or corrective saccades, since those were rejected by our 90% criterion (see Method; see also Unema et al., 2005). They also are unlikely to represent inspections of the space between landmark and door, since that space contained little visual details. We rather assume that interim fixations were programmed such as to avoid large-amplitude saccades. Indeed, the shortest distance between the edges of landmarks and doors in our study subtended a visual angle of about 9°, which is near the top end of the saccadic amplitude range during visual scene inspections (Tatler et al., 2006; Unema et al., 2005).

Although our data provided no evidence for a gradual gaze shift during decision-making in a wayfinding task, this conclusion is necessarily limited to the particular task under study. It therefore remains conceivable that a gradual shift might emerge if the wayfinding decisions are more difficult, e.g., if participants must choose between six instead of three doors, or if they must search for the doors within a cluttered, realistic scene that includes objects like tables, chairs, cupboards, rugs, and lamps. It has indeed been shown that participants search for a particular object in such cluttered scenes by executing first a large saccade into the general area where they expect that object to be, and then one or several smaller saccades until they capture the object with their gaze (Neider & Zelinsky, 2006; Wolfe, 2021; Zhang et al., 2018). These gradual gaze shifts have been attributed to top-down processes that guide the viewer’s attention, but the relationship between gaze control, deployment of attention and decision-making still remains to be elucidated. As an example, future research could compare decision-making with restrained and with free gaze, to determine whether gaze shifts are an integral component or rather a byproduct of decision-making.

## Data Availability

The datasets generated during and/or analyzed during the current study are available from the corresponding author on reasonable request.

## Code availability

The scripts for generating the task in the current study are available from the corresponding author on reasonable request.
